# Low pH Attenuates Apoptosis by Suppressing the Volume-Sensitive Outwardly Rectifying (VSOR) Chloride Current in Chondrocytes

**DOI:** 10.3389/fcell.2021.804105

**Published:** 2022-02-02

**Authors:** Michael Kittl, Martina Winklmayr, Julia Preishuber-Pflügl, Victoria Strobl, Martin Gaisberger, Markus Ritter, Martin Jakab

**Affiliations:** ^1^ Center for Physiology, Pathophysiology and Biophysics, Institute for Physiology and Pathophysiology—Salzburg, Paracelsus Medical University, Salzburg, Austria; ^2^ Ludwig Boltzmann Institute for Arthritis and Rehabilitation, Salzburg, Austria; ^3^ Gastein Research Institute, Paracelsus Medical University, Salzburg, Austria; ^4^ Center for Physiology, Pathophysiology and Biophysics, Institute for Physiology, Pathophysiology and Biophysics—Nuremberg, Paracelsus Medical University, Nuremberg, Germany

**Keywords:** apoptosis, AVD, caspase, chloride, chondrocytes, pH, viability, volume regulation

## Abstract

In a variety of physiological and pathophysiological conditions, cells are exposed to acidic environments. Severe synovial fluid acidification also occurs in a progressive state of osteoarthritis (OA) affecting articular chondrocytes. In prior studies extracellular acidification has been shown to protect cells from apoptosis but the underlying mechanisms remain elusive. In the present study, we demonstrate that the inhibition of Cl^−^ currents plays a significant role in the antiapoptotic effect of acidification in human articular chondrocytes. Drug-induced apoptosis was analyzed after exposure to staurosporine by caspase 3/7 activity and by annexin-V/7-actinomycin D (7-AAD) staining, followed by flow cytometry. Cell viability was assessed by resazurin, CellTiter-Glo and CellTiter-Fluor assays. Cl^−^ currents and the mean cell volume were determined using the whole cell patch clamp technique and the Coulter method, respectively. The results reveal that in C28/I2 cells extracellular acidification decreases caspase 3/7 activity, enhances cell viability following staurosporine treatment and gradually deactivates the volume-sensitive outwardly rectifying (VSOR) Cl^−^ current. Furthermore, the regulatory volume decrease (RVD) as well as the apoptotic volume decrease (ADV), which represents an early event during apoptosis, were absent under acidic conditions after hypotonicity-induced cell swelling and staurosporine-induced apoptosis, respectively. Like acidosis, the VSOR Cl^−^ current inhibitor DIDS rescued chondrocytes from apoptotic cell death and suppressed AVD after induction of apoptosis with staurosporine. Similar to acidosis and DIDS, the VSOR channel blockers NPPB, niflumic acid (NFA) and DCPIB attenuated the staurosporine-induced AVD. NPPB and NFA also suppressed staurosporine-induced caspase 3/7 activation, while DCPIB and Tamoxifen showed cytotoxic effects *per se*. From these data, we conclude that the deactivation of VSOR Cl^−^ currents impairs cell volume regulation under acidic conditions, which is likely to play an important role in the survivability of human articular chondrocytes.

## Introduction

The volume-sensitive outwardly rectifying (VSOR) Cl^−^ channel is ubiquitously expressed in almost all vertebrate cells and is a key player in cell volume regulatory processes. The channel, which opens upon osmotic cell swelling, contributes to regulatory volume decrease (RVD) by extruding anions and organic osmolytes (Jentsch, 2016; Pedersen et al., 2016; Chen et al., 2019; Okada et al., 2020). In addition to its volume regulatory role in various physiological functions like migration, phagocytosis, and proliferation (Jakab and Ritter, 2006; Schwab et al., 2012; Harl et al., 2013; Wanitchakool et al., 2016; Kittl et al., 2019b), VSOR anion channels are also involved in apoptotic cell death (Okada and Maeno, 2001; Okada et al., 2006; Kondratskyi et al., 2015; Pedersen et al., 2016; Okada et al., 2020).

Apoptosis is characterized by a normotonic cell shrinkage that occurs at an early phase followed by caspase activation, chromatin condensation, DNA fragmentation and plasma membrane blebbing (Elmore, 2007). This initial normotonic cell shrinkage, referred to as apoptotic volume decrease (AVD), is a major hallmark of apoptosis and is necessary to induce apoptotic cell death (Maeno et al., 2000; Okada and Maeno, 2001; Bortner and Cidlowski, 2004; Ernest et al., 2008; Poulsen et al., 2010; Kondratskyi et al., 2015; Okada et al., 2020). Several authors concluded that the activation of VSOR channels is involved in AVD by showing that on the one hand inducers of apoptosis like staurosporine, doxorubicin, FAS ligand, TNFα, or H_2_O_2_ activate VSOR channels under normotonic conditions (D’anglemont De Tassigny et al., 2004; Porcelli et al., 2004; Shimizu et al., 2004; Jiao et al., 2006; Okada et al., 2020) and on the other hand pharmacological inhibition of VSOR channels impairs apoptosis by preventing AVD (Maeno et al., 2000; Okada et al., 2006). Not only pharmacological inhibition but also LRRC8 knock-out cells, which were lacking either the LRRC8A isoform or all LRRC8 isoforms, displayed reduced caspase-3 activity after exposure to staurosporine or cisplatin (Planells-Cases et al., 2015). Moreover, a facilitated RVD response under hypotonic conditions in apoptotic cells indicates, that both mechanisms—AVD and RVD—are coupled and require the activation of VSOR channels to induce normotonic or hypotonic cell shrinkage, respectively. Additionally, the assumption of a common mechanism underlying both AVD and RVD is supported by the finding that AVD induction as well as RVD facilitation are prevented by the application of the same Cl^−^ channel blockers (Maeno et al., 2000; Okada and Maeno, 2001; Okada et al., 2001; Okada et al., 2020). While shrunken cells under physiological conditions undergo a regulatory volume increase (RVI) by gain of solutes and water (Lang et al., 1998; Hoffmann et al., 2009), apoptotic cells remain shrunken without compensating the cell volume (Maeno et al., 2006). This indicates that cell volume regulatory processes, like RVI and RVD, which “normally” protect cells from excessive cell shrinkage or cell swelling, respectively, operate differently in apoptotic cells. In this regard, RVD is supposed to be activated under normotonic conditions to induce apoptosis (AVD) and RVI might be overridden to preserve cell shrinkage during apoptosis (Bortner and Cidlowski, 1996; Maeno et al., 2000). In recent studies we could show that lowering the extracellular pH to ≤5.0 leads to a deactivation of the VSOR current and to an impaired volume regulation in microglial cells and chondrocytes (Kittl et al., 2019a; Kittl et al., 2020). Acidity is also reported to influence apoptosis, but literature data are contradictory. While several studies have demonstrated that a low extracellular pH favors the induction of apoptosis (Ding et al., 2000; Thatte et al., 2004; Kumar et al., 2007), in other studies acidification has been found to inhibit apoptosis. A protective effect of acidosis has been observed in different cell types including endothelial cells (D’Arcangelo et al., 2000; D’Arcangelo et al., 2002; Terminella et al., 2002; Kumar et al., 2008), liver cells (Currin et al., 1991), lymphoblastic cells (Bohloli et al., 2016), neurons (Xu et al., 1998), colon adenocarcinoma cells (Sharma et al., 2005) and osteoclasts (Pereverzev et al., 2008). The mechanisms underlying the protective effect of acidosis against apoptosis are still unclear.

Our present study provides more insight into this topic by investigating the effect of acidosis on apoptosis in human articular chondrocytes and testing whether VSOR Cl^−^ channels play a role in the capability of acidosis to inhibit apoptosis.

## Materials and Methods

### Salts, Chemicals, Drugs

All salts and chemicals were p.a. grade. DCPIB (4-[(2-butyl-6,7-dichloro-2-cyclopentyl-1-oxo-3H-inden-5-yl)oxy]butanoic acid) was purchased from Tocris (Abingdon, United Kingdom), DIDS (4,4′-diisothiocyano-2,2′-stilbenedisulfonic acid), niflumic acid (NFA; 2-[3-(trifluoromethyl)anilino]pyridine-3-carboxylic acid), NPPB (5-nitro-2-(3-phenylpropylamino)benzoic acid), tamoxifen (2-[4-[(Z)-1,2-diphenylbut-1-enyl]phenoxy]-N,N-dimethylethanamine) and staurosporine were purchased from SigmaAldrich-Merck (Darmstadt, Germany). Stock solutions of DCPIB (100 mM) and Tamoxifen (40 mM) were prepared in ethanol. DIDS, NFA, NPPB and staurosporine were dissolved in dimethyl sulfoxide (DMSO) to give stock solutions of 100 mM, 100 mM, 100 mM and 1 mM, respectively. The stocks were stored in aliquots at −20°C until use.

### Cell Culture

Human immortalized C28/I2 cells, were cultured in 25 cm^2^ flasks with DMEM/HAM’s F-12 medium (Biochrom, Berlin, Germany) supplemented with 5% fetal bovine serum (FBS Superior, Biochrom) and antibiotic-antimycotic solution (100 U/ml penicillin, 0.1 mg/ml streptomycin, 0.25 μg/ml amphotericin-B; Sigma-Aldrich-Merck). C28/I2 cells were kept at 37°C in a humified atmosphere of 5% CO_2_ (standard culture conditions). Subcultures were established once a week until passage 25.

### Acidification Protocol

To expose C28/I2 cells to different pH conditions during culture, different cell culture media were prepared as shown in Table 1. Briefly, 8.3 g of Dulbecco’s Modified Eagle’s Medium (DMEM) D5030-1L (Sigma-Aldrich-Merck) powder was dissolved in distilled water and supplemented with D-glucose (3.15 g/l), sodium-pyruvate (0.06 g/l) and L-glutamine (0.37 g/l). NaHCO_3_ was added according to the required pH. For acidic media (pH ≤ 6.6) MES free acid (FA) was used instead of HEPES FA. The osmolality was adjusted by the addition of NaCl. The pH, which was measured in the incubator under standard culture conditions, achieved a stable value approximately after 2 h and remained constant for at least 24 h.

**TABLE 1:** Media composition. Concentrations in mM, osmolality in mOsm/kg.

pH	7.4	6.6	6.0	5.5	5.2
NaHCO_3_^−^	25.0	7.5	3.5	1.5	0.8
HEPES-FA	5.0	0.0	0.0	0.0	0.0
MES-FA	0.0	5.0	5.0	5.0	5.0
NaCl	0.0	14.0	15.0	15.0	15.0
Osmolality	299.0	300.0	299.0	300.0	303.0

### Patch Clamp

C28/I2 cells were seeded on 0.01% poly-D-lysine (PDL)-coated coverslips (12 mm diameter) and cultured for at least 24 h in DMEM/HAM’s F-12 medium. Coverslips were transferred to a RC-25 recording chamber (Warner Instruments, Hamden, CT, United States) and mounted on a Nikon Eclipse TE2000-U inverted microscope. Experiments were performed at room temperature in the whole cell perforated patch clamp mode adding 130 µM amphotericin to the pipette solution. Recordings were started as soon as the serial resistance was below 30 MΩ. The resistances of the patch electrode were between 4 and 9 MΩ. After establishing the whole-cell configuration, cells were superfused with an extracellular solution and data were recorded using an EPC-10 amplifier controlled by PatchMaster software (HEKA, Lambrecht/Pfalz, Germany). Voltage clamp recordings of Cl^−^ currents under neutral and acidic conditions were performed under symmetrical intra- and extracellular Cl^−^ conditions. The extracellular solution consisted of (in mM): NaCl 100, CaCl_2_ 2.5, MgCl_2_ 2.5, HEPES FA 10 and mannitol 90 (300 mOsm/kg, pH 7.2 adjusted with NaOH). In our patch clamp experiments we did not use MES to buffer the pH, because the relatively high flow rate of the perfusion system (3–5 ml/min) apparently prevents significant pH deviations in the proximity of the cell. Mannitol was omitted to obtain a hypotonic (220 mOsm/kg) solution for VSOR current activation. To assess pH dependent effects, the extracellular solution was titrated with HCl to a pH of 6.0. The pipette solution contained (in mM): CsCl 100, MgCl_2_ 5, HEPES FA 10, EGTA 11, raffinose 60, Mg-ATP 2 (303 mOsm/kg, pH 7.2 adjusted with CsOH). The currents were monitored in response to voltage ramps (500 ms duration, 10-s intervals) and voltage steps (500 ms duration, increments of 20 mV) from −100 mV to +100 mV. The holding potential between the ramps/steps was 0 mV to desensitize voltage-activated currents. Bath solution exchange was performed with a valve-controlled gravity-driven perfusion system (ALA Scientific Instruments, Farmingdale, NY, United States) at a flow rate of 3–5 ml/min.

### Cell Volume Measurements

**FIGURE 1:**
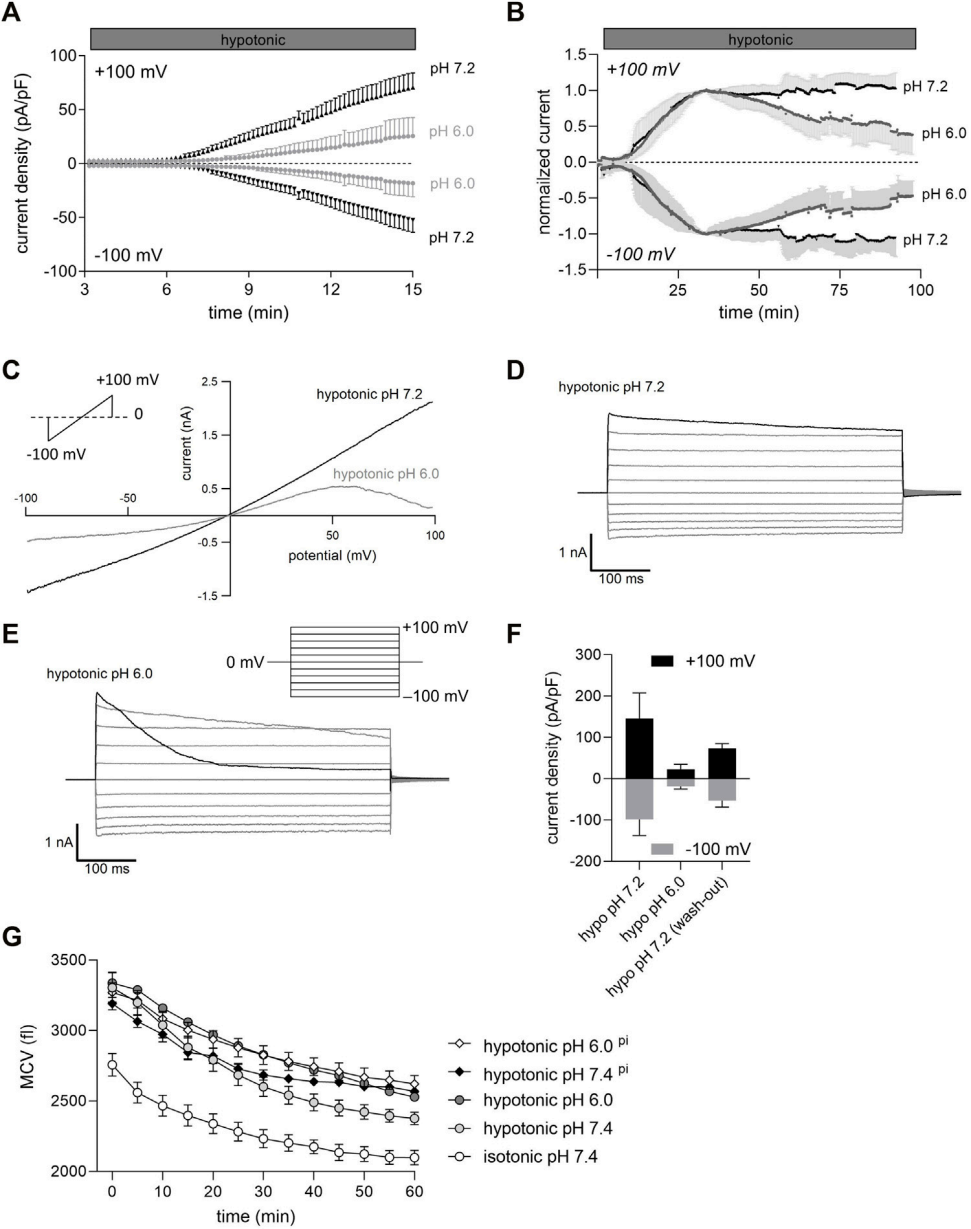
Extracellular acidification inhibits VSOR Cl^−^ currents and impairs RVD in C28/I2 cells. **(A)** Mean Cl^−^ current ± SEM measured over 15 min under pH 7.2 and pH 6.0 conditions. 24 h before Cl^−^ currents were measured chondrocytes were preincubated in pH 7.4 medium (control, dark grey triangles), or in pH 6.0 medium (light grey circles). Each symbol represents the current normalized to cell membrane capacity (current density), which was obtained every 10 s at +100 mV (upper traces) and −100 mV (lower traces) in response to 500-ms voltage ramps applied every 10 s from a holding potential of 0 mV (n = 6–8). **(B)** Time course of mean Cl^−^ current amplitudes under hypotonic pH 7.2 and pH 6.0 conditions. pH 6.0 solution was applied when Cl^−^ currents reached a maximum under hypotonic conditions. Currents were normalized to peak values under hypotonic conditions. Each circle represents the current at +100 mV (upper traces) and −100 mV (lower traces) measured in response to 500-ms voltage ramps applied every 10 s from a holding potential of 0 mV (n = 6–7). **(C)** Representative current voltage relationship (500-s voltage ramps applied from −100 mV to +100 mV) recorded under hypotonic pH 7.2 and hypotonic pH 6.0 conditions. **(D)** and **(E)** Representative VSOR Cl^−^ current traces elicited by 500-ms voltage steps from −100 mV to +100 mV in 20-mV increments from a holding potential of 0 mV, obtained at pH 7.2 and pH 6.0 as indicated. **(F)** Peak current densities (pA/pF) at +100 mV and −100 mV under hypotonic conditions (hypo pH 7.2), after 75–100 min at hypotonic pH 6.0 (hypo pH 6.0) and after reapplication of hypo pH 7.2 solution (wash-out). **(G)** Mean cell volume (MCV) ± SEM in femtoliters (fl) measured over 1 h under isotonic (300 mOsm/kg) or hypotonic (220 mOsm/kg) conditions at pH 7.4 or pH 6.0 (n = 4–8). For two conditions—hypotonic pH 7.4 ^pi^ and hypotonic pH 6.0 ^pi^ (diamonds)—cells were preincubated in an acidic medium (pH 6.0) for 24 h. For the other conditions - isotonic pH 7.4, hypotonic pH 7.4 and hypotonic pH 6.0 (circles), cells were incubated with the same media but under pH 7.4.

C28/I2 cells were harvested by Trypsin/EDTA after growing under standard conditions. The cell suspension was split into aliquots, which were centrifuged for 4 min at 200×g. The supernatants were discarded. Immediately before the first measurement (time point 0) the cell pellet was re-suspended in 20 ml of an extracellular solution. For experiments shown in Figure 1G, the solution contained (in mM): NaCl 100, KCl 5.6, CaCl_2_ 2.5, MgCl_2_ 1.5, HEPES FA 10, MES FA 5, glucose 4.5, and mannitol 80 (300 mOsm/kg). Mannitol was omitted to obtain a hypotonic (220 mOsm/kg) solution. For experiments shown in Figures 7A–F, the extracellular solution contained 140 mM NaCl without mannitol (300 mOsm/kg). A hypertonic extracellular solution (360 mOsm/kg) was obtained by the addition of mannitol. The pH of the extracellular solution was adjusted to 7.4 or 6.0 with NaOH or HCl, respectively. VSOR Cl^−^ current inhibitors (NPPB, DCPIB, niflumic acid (NFA), Tamoxifen and DIDS) and the apoptosis inducer staurosporine, which was used to induce AVD, were added to the extracellular solutions as indicated in the individual experiments. Samples in the absence of VSOR Cl^−^ inhibitors and staurosporine were complemented by a corresponding amount of the solvent (DMSO). The mean cell volumes (MCV in femtoliters) in the different samples were alternately measured on a Beckman Coulter Z2 particle counter (Beckman Coulter, Krefeld, Germany). In the first set of experiments (Figure 1G) samples were measured every 5 min for 60 min, in the experiments shown in Figure 7A samples were measured every 5 min for the first 15 min and every 15 min for the next 105 min and in the experiments depicted in Figures 7C,E samples were measured every 10 min for 60 min. The principle of the measurement is based on changes in electrical resistance produced by nonconductive particles suspended in an electrolyte solution (Coulter method). Calibration for particle size was performed using 10 µm Flow-Check fluorospheres (Beckman-Coulter). Data were analyzed with the Multisizer Software (Beckman Coulter) using a 600-fl cutoff to exclude cell debris.

### Cell Viability Assays

C28/I2 cells were seeded at a density of 1×10^4^ cells per well into a transparent 96-well microplate (CytoOne; Starlab, Hamburg, Germany). After 24 h, the medium was replaced by serum-free medium (-FBS) and cells were grown for another 24 h before treatment. For treatment, the medium was replaced by media with different pH values, VSOR Cl^−^ current inhibitors and staurosporine concentrations, as indicated in the individual experiments. Samples in the absence of VSOR Cl^−^ channel inhibitors and staurosporine were complemented by a corresponding amount of the solvent (DMSO). After a treatment period of 8 and 24 h (incubation), cell viability was measured in a 96-well microplate using the resazurin (7-Hydroxy-3H-phenoxazin-3-one-10-oxide sodium salt; Sigma-Aldrich-Merck) assay, CellTiter-Fluor assay or the CellTiter-Glo assay (Promega, Mannheim, Germany). For the resazurin assay the culture supernatant was removed and replaced by 100 µl serum-free medium containing 0.5 mM resazurin (stock solution 2.5 mM in PBS). After the incubation of 1 h at 37°C and 5% CO_2_ the viability was measured by detecting the fluorescence of the product (resorufin) at λ_ex_ = 535 nm and λ_em_ = 595 nm using a Tecan Spark microplate reader (Tecan, Grödig, Austria). CellTiter-Fluor and the CellTiter-Glo assays were performed according to the manufacturer’s protocols. Fluorescence (CellTiter-Fluor; λ_ex_ = 380 nm and λ_em_ = 505 nm) and luminescence (CellTiter-Glo) were measured using a Tecan Spark microplate Reader. Mean viability values were corrected for blank values (without cells). All treatments were measured at least in triplicate wells.

### Caspase 3/7 Activity

Like for cell viability assays, 1×10^4^ cells per well were seeded into a transparent 96-well microplate (CytoOne). After 24 h, the medium was replaced by serum-free medium, and cells were grown for another 24 h before treatment. After two different treatment periods of 8 and 24 h, 50 µl of Caspase-Glo 3/7 assay substrate (Promega) was added and cells were incubated for another 30 min. Then the well contents were transferred to a white-walled 96-well plate (Greiner bio-one, Germany). Luminescence was measured in a Tecan Spark multimode reader (Tecan). All treatments were measured at least in triplicates.

### Annexin-V/7-Actinomycin D Staining

For assessment of phosphatidylserine exposure at the cell surface by annexin-V binding and cell membrane integrity by 7-AAD staining, 1.5×10^5^ cells were seeded in 35 mm diameter Petri dishes. After a cell cultivation period of 24 h under standard conditions the medium was changed to serum-free medium (-FBS). The following day, cells were incubated for 5 h under different pH conditions and staurosporine concentrations, as indicated in the individual experiments. Samples in the absence of staurosporine were treated with an equivalent amount of solvent (DMSO). Thereafter, the cells were harvested and the annexin-V/7-AAD assay was performed following the manufacturer’s protocol (BioLegend, San Diego, CA, United States) and measured by flow cytometry on a Cell Lab Quanta SC flow cytometer (Beckman Coulter). Data were analyzed using the FLOWJO software (Becton, Dickinson and Company; Franklin Lakes, NJ, United States).

### Data Presentation

Data are expressed as means ± standard error of the means (SEM) of at least three independent biological replicates (n ≥ 3). In all experimental series, solvent control samples were included. Data were analyzed and plotted using GraphPad Prism 9 (GraphPad Software, La Jolla, CA, United States) or Igor Pro 8 (WaveMetrics, Portland, OR, United States).

## Results

### Extracellular Acidification Affects the Functional Activity of Volume-Sensitive Outwardly Rectifying Cl^−^ Channels and Impairs Cell Volume Regulation in Chondrocytes

In most cells including chondrocytes, cell swelling leads to the activation of distinct anion channels (VSOR channels) and gives rise to a typical swelling activated Cl^−^ current (ICl_swell_). In C28/I2 cells under hypotonic conditions the current slowly developed over time at pH 7.2 (Figure 1A) and the current-voltage relationship revealed a moderate outward rectification and a (slight) time-dependent inactivation at constant positive holding potentials (+100 mV) (Figure 1D). Time-dependent inactivation was more pronounced shortly after exposing cells to an acidic pH of 6.0 (Figure 1E). Continued exposure to pH 6.0 and hypotonic conditions led to a progressive decline in the current amplitude until the current was almost abolished after 90 min (Figures 1B,C). Preincubation for 24 h of C28/I2 cells under acidic (pH 6.0) conditions prior to a hypotonic pH 6.0 challenge decreased the Cl^−^ current amplitude after 15 min by ∼75% and delayed the onset of current activation by ∼3 min, as compared to control conditions (Figure 1A). As shown in Figure 1F, perfusion of cells with hypotonic pH 7.2 extracellular solution after 75–100 min of current deactivation under pH 6.0 led to ∼40% current recovery. Considering that VSOR currents are crucial for regulating the cell volume, by extruding anions and/or organic osmolytes and osmotically obliged water to counteract osmotic cell swelling we performed cell volume measurements in C28/I2 cells under isotonic, hypotonic, and acidic conditions to test whether there is a difference in the cells’ volume regulating ability at low pH. Under isotonic pH 7.4 (control) conditions we observed a moderate cell shrinkage of ∼24% from a mean cell volume (MCV) of ∼2,750 fl to ∼2,100 fl over 60 min (Figure 1G). Under hypotonic pH 7.4 conditions the MCV at the first timepoint (0 min) was ∼550 fl higher than under isotonic conditions. Over 60 min this difference in MCV progressively decreased to ∼250 fl, indicative for RVD. In cells exposed to an acidic and hypotonic (hypotonic pH 6.0) condition, the difference in MCV to isotonic conditions remained constant over 60 min, indicating a missing RVD response under low pH. Similarly, a 24 h preincubation of cells at pH 6.0 (indicated as ^pi^), impaired the RVD under hypotonic pH 6.0 conditions and under hypotonic pH 7.4 (Figure 1G).

### Pharmacological Properties of Volume-Sensitive Outwardly Rectifying Cl^−^ Channels

**FIGURE 2:**
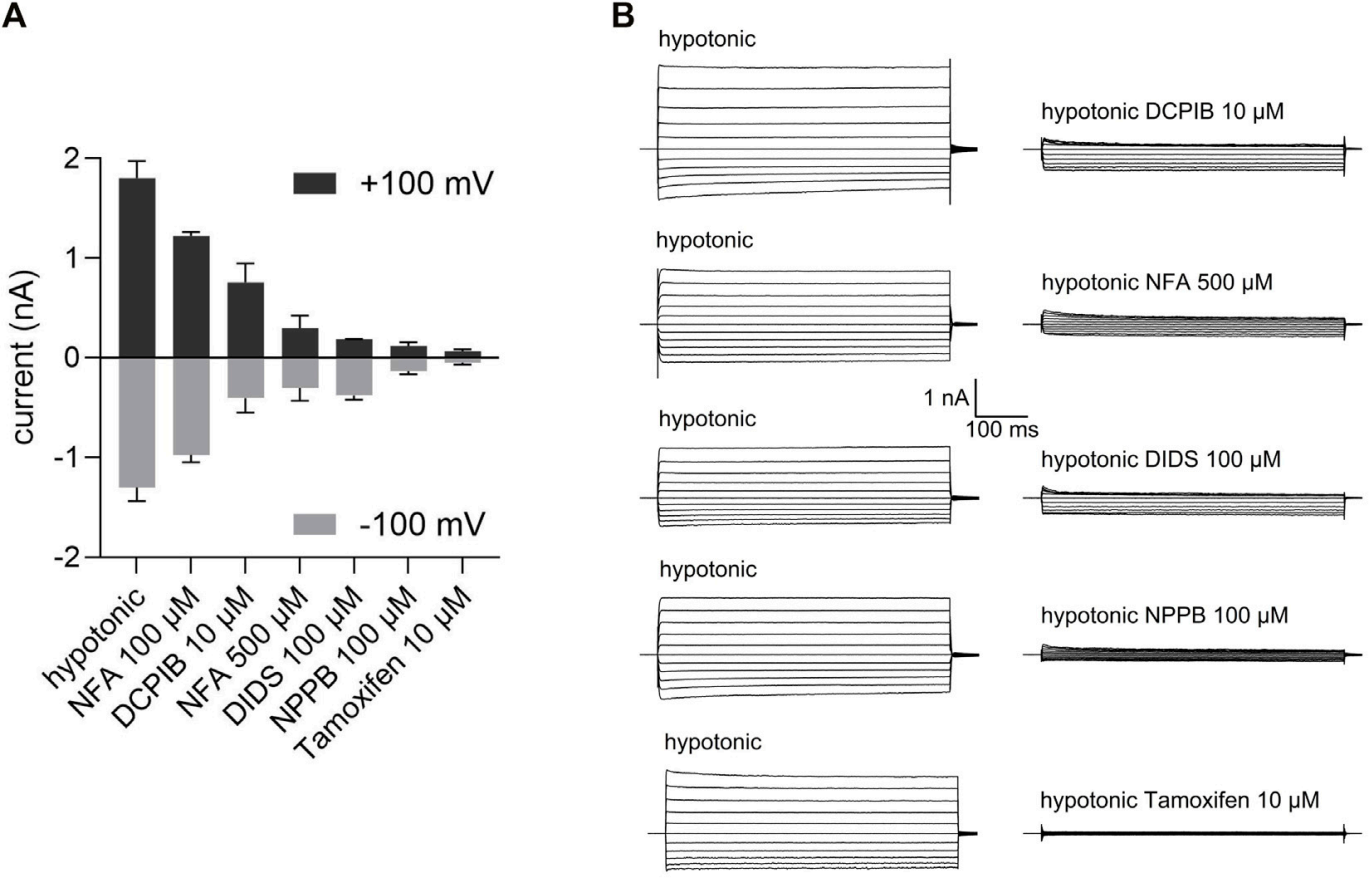
Pharmacological properties of VSOR Cl^−^ channels in C28/I2 cells. **(A)** Mean peak VSOR Cl^−^ current amplitudes ± SEM at +100 mV and −100 mV under hypotonic conditions in the absence and presence of different inhibitors. **(B)** VSOR Cl^−^ current traces elicited by 500-ms voltage steps from −100 mV to +100 mV obtained at maximal VSOR Cl^−^ current amplitudes under hypotonic conditions in the absence and presence of DCPIB (10 µM), niflumic acid (NFA; 500 µM), DIDS (100 µM), NPPB (100 µM) and Tamoxifen (10 µM) (n = 3).

We quantified the sensitivity of the VSOR Cl^−^ current to five different Cl^−^ channel inhibitors—niflumic acid (NFA), DCPIB, DIDS, NPPB and Tamoxifen. To this end VSOR Cl^−^ currents were activated by 80 mOsm/kg reduction in extracellular osmolality until a plateau was reached. As shown in Figures 2A,B the most selective and potent VSOR channel blockers in C28/I2 cells were Tamoxifen and NPPB, which almost fully blocked the inward and the outward current at concentrations of 10 and 100 μM, respectively. DIDS at 100 µM inhibited the outward current at +100 mV to 13 ± 3% similar to Tamoxifen and NPPB, while the inward current at −100 mV was less sensitive to DIDS with an inhibition to 39 ± 13%. At a concentration of 500 µM niflumic acid (NFA) potently inhibited the inward and outward Cl^−^ current to 15 ± 5% and 18 ± 7%, respectively, while at a concentration of 100 μM, NFA was insufficient to inhibit the current. DCPIB, which is regarded as a strong and selective VSOR channel blocker (Pedersen et al., 2016; Friard et al., 2017; Okada et al., 2019), was far less potent in chondrocytes compared to the other inhibitors tested, showing an inhibition to 35 ± 5% at +100 mV and 30 ± 4% at −100 mV.

### Acidic Preconditioning Protects Chondrocytes Against Staurosporine-Induced Apoptosis

**FIGURE 3:**
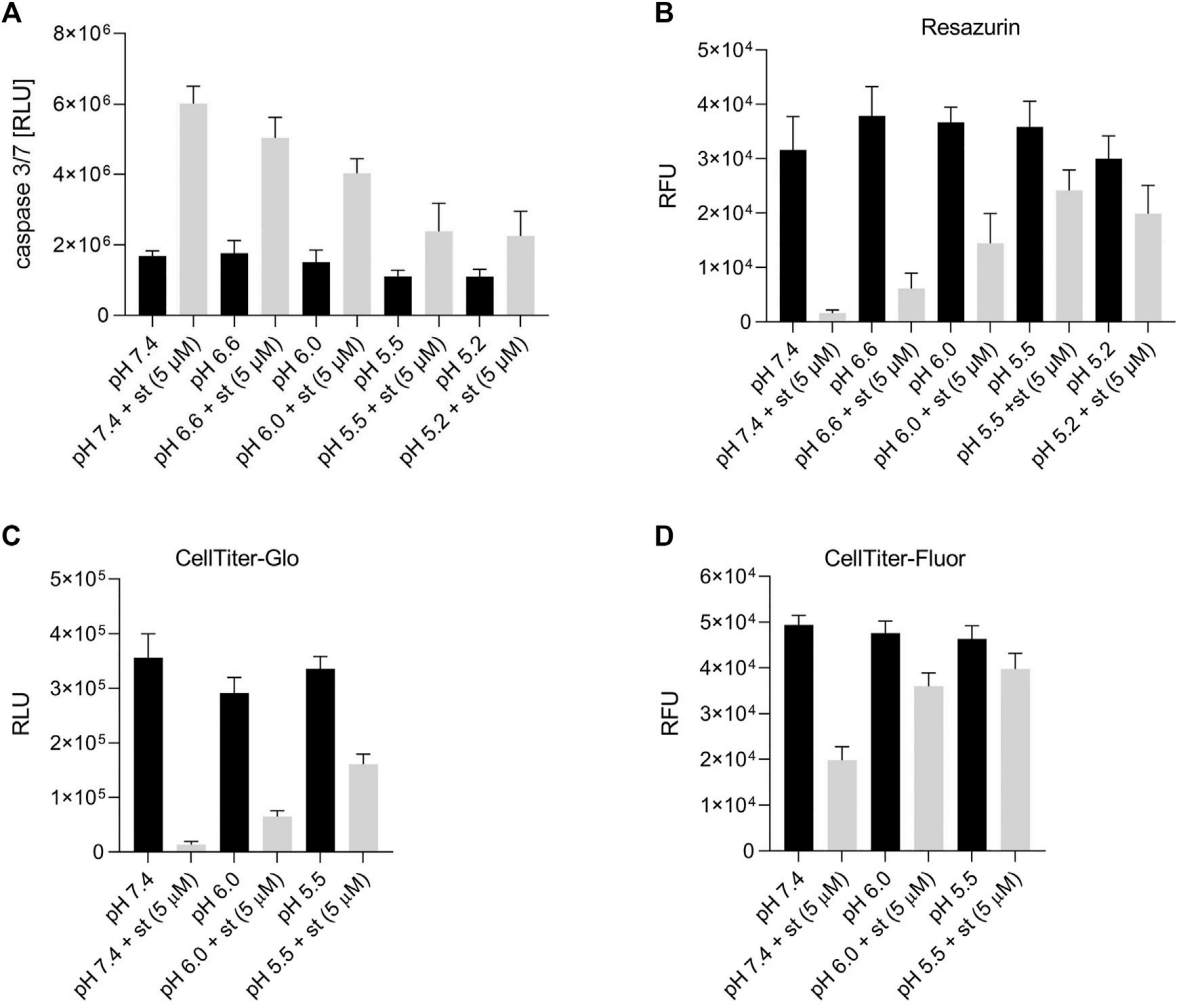
Effect of acidic preconditioning on caspase 3/7 activity and cell viability. **(A)** Caspase 3/7 activity and viability assessed by **(B)** resazurin and **(C)** CellTiter-Glo assays based on the cells’ metabolic activity and ATP content and **(D)** CellTiter-Fluor assays based on constitutive protease activity in living cells. C28/I2 cells were kept for 8 h at different pH values in the absence or presence of staurosporine (st) (5 µM) (n = 3).

In a next set of experiments, we examined the effect of different extracellular pH (7.4, 6.6, 6.0, 5.5 and 5.2) on caspase 3/7 activity after treatment with the apoptosis inducer staurosporine (st) (5 µM). Under control conditions (pH 7.4) in the absence of staurosporine, a low caspase 3/7 activity was measured in C28/I2 cells, which slightly decreased, when cells were incubated for 8 h in acidic media (pH 6.0, 5.5 and 5.2) (Figure 3A). Exposure to 5 µM staurosporine for 8 h elicited a pH-dependent 1.5–3fold increase in the caspase 3/7 activity. The maximum of caspase 3/7 activity was observed under pH 7.4 conditions after staurosporine treatment, which decreased progressively with decreasing pH (6.6, 6.0, 5.5 and 5.2). Cell viability was assessed by resazurin, CellTiter-Glo and CellTiter-Fluor assays. The CellTiter-Fluor assay measures a constitutive protease activity within living cells whereas resazurin and CellTiter-Glo assays are based on the quantitation of ATP present in metabolically active cells. For all three methods the results were similar. In resazurin assays the cell viability within 8 h of treatment with 5 µM staurosporine dropped in a pH-dependent manner, with a strong reduction in the viability at pH 7.4 and progressively less reduction in cell viability from pH 6.6 to pH 5.5 and 5.2 (Figure 3B). Viability was ∼5% under pH 7.4 plus 5 µM staurosporine, while it was over 65% in low pH media (5.5 and 5.2), compared to their controls in the absence of staurosporine (Figure 3B). The effect was similar in CellTiter-Glo assays showing ∼48% viability under pH 5.5 in the presence of staurosporine compared to ∼4% at pH 7.4 + st (Figure 3C). Compared with resazurin and CellTiter-Glo assays, in CellTiter-Fluor measurement the ratio of viable cells was higher in presence of staurosporine compared to the respective controls (∼40%, 56% and ∼86% at pH 7.4, 6.0 and 5.5, respectively) (Figure 3D). This is probably because assessment of cell viability *via* protease activity using CellTiter-Fluor more selectively reflects the actual number of living cells, while the signal in resazurin and CellTiter-Glo assays is not only proportional to the absolute cell number but also to the cells’ metabolic activity and ATP content.

**FIGURE 4:**
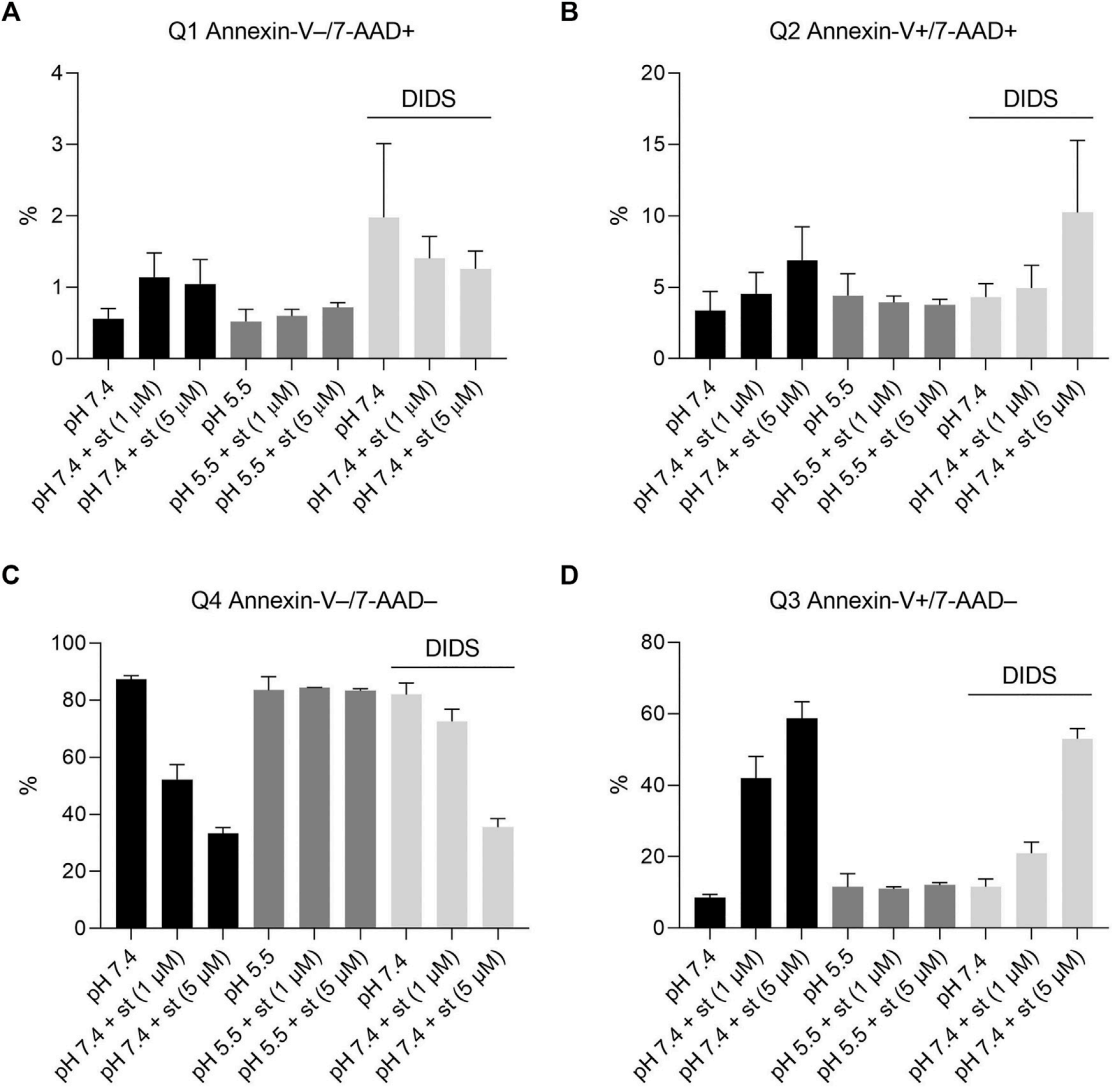
Effect of acidic preconditioning on annexin V/7-AAD staining. Annexin V/7-AAD staining of C28/I2 cells after 5 h incubation under neutral pH 7.4, acidic pH 5.5 and neutral pH 7.4 + DIDS (100 µM) conditions in the absence and presence of staurosporine (st) (1 and 5 µM). **(A)** Q1 annexin-V–/7-AAD+ (necrotic cells) **(B)** Q2 annexin-V+/7-AAD+ (late apoptotic cells) **(C)** Q4 annexin-V−/7-AAD− (living cells), **(D)** Q3 annexin-V+/7-AAD− (early apoptotic cells) (n = 4).

**FIGURE 5:**
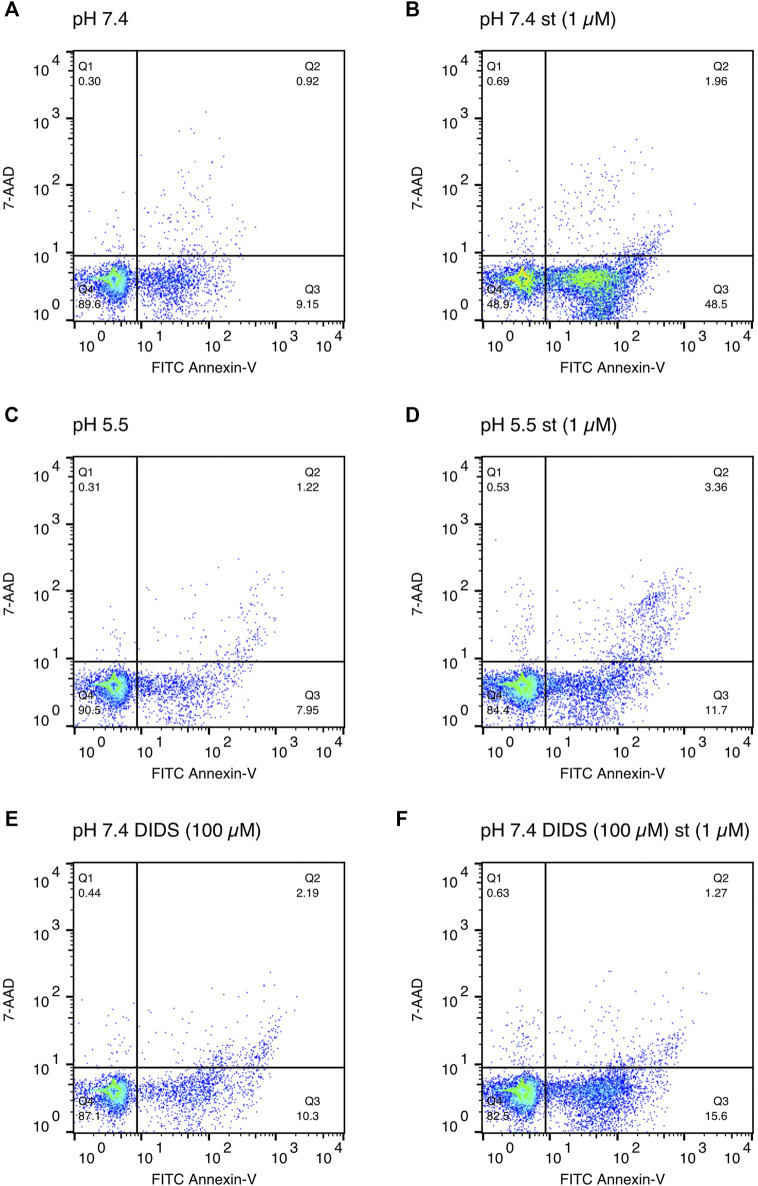
Exemplary annexin V/7-AAD staining scatter plots after 5 h of incubation in media of **(A)** pH 7.4, **(B)** pH 7.4 with staurosporine (st) (1 µM), **(C)** pH 5.5, **(D)** pH 5.5 with staurosporine (1 µM), **(E)** pH 7.4 in presence of DIDS (100 µM) and **(F)** pH 7.4 with staurosporine in presence of DIDS (100 µM).

Annexin-V/7-AAD double staining was performed to confirm apoptosis. After a 5-h treatment with 1 and 5 µM staurosporine under pH 7.4 conditions, there was a decline in the Q4 annexin-V–/7-AAD– (living) cell population accompanied by a 5fold (1 µM staurosporine) and 6.5fold (5 µM staurosporine) increase in the Q3 annexin-V+/7-AAD– (early apoptotic) population (Figures 4C,D and Figures 5A,B). This staurosporine-induced decline in the Q4 annexin-V–/7-AAD– (living) cell population and increase in the Q3 annexin-V+/7-AAD– (early apoptotic) cell population was reversed under acidic conditions (pH 5.5), showing ∼85% living cells and ∼10% early apoptotic cells, which was similar to control conditions (pH 7.4 without st) (Figures 4C,D and Figures 5C,D). The effects of staurosporine and acidification were less pronounced in the Q2 Annexin-V+/7-AD+ (late apoptotic) and Q1 annexin-V–/7-AAD+ (necrotic) cell populations (Figures 4A,B and Figures 5A–D).

**FIGURE 6:**
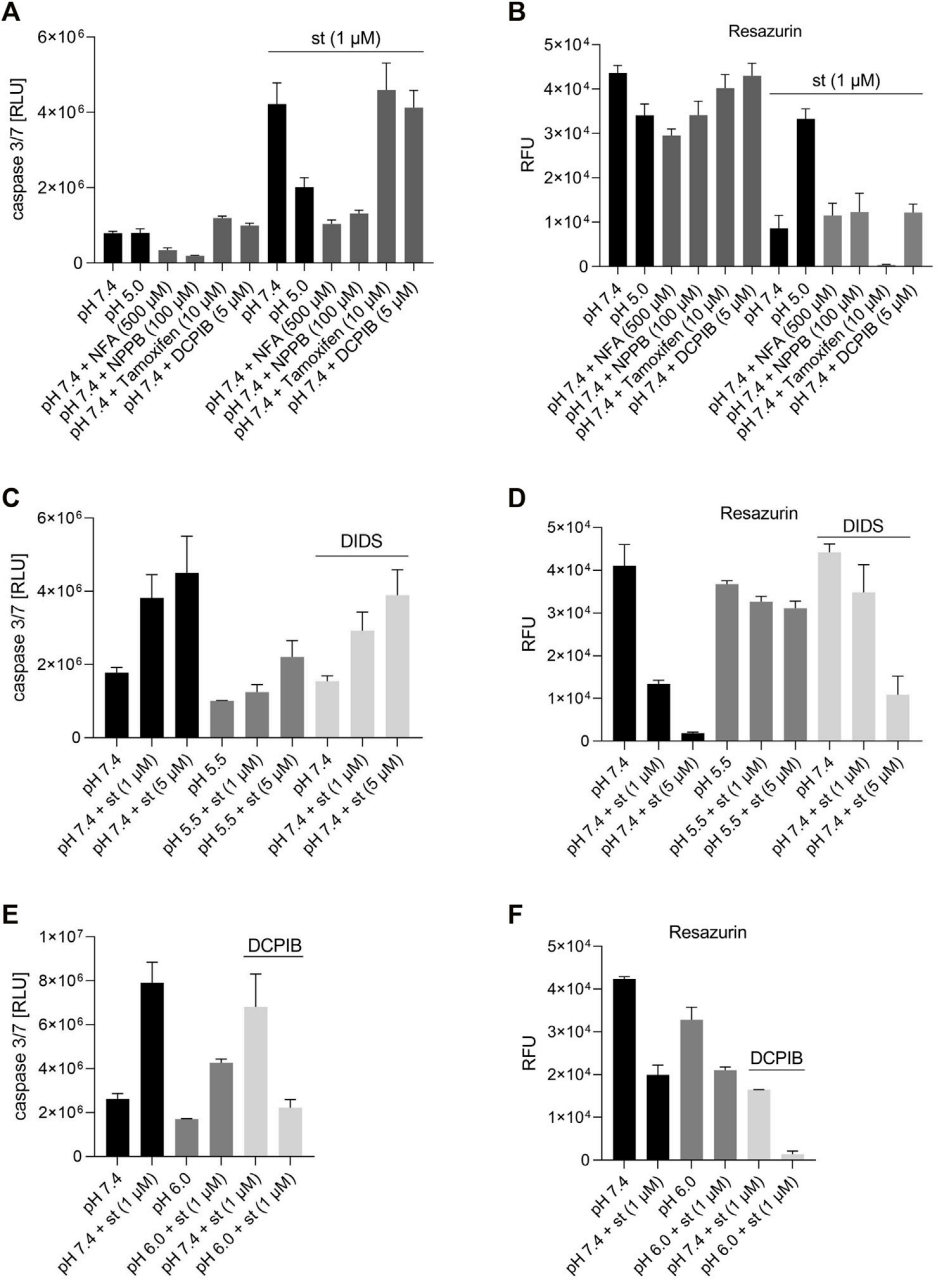
Effect of different VSOR Cl^−^ channel inhibitors on caspase 3/7 activity and cell viability. **(A)** Caspase 3/7 activity and **(B)** viability of C28/I2 cells treated for 6 h with pH 7.4, pH 5.5 and pH 7.4 + different VSOR Cl^−^ current inhibitors in the absence and presence of staurosporine (st) (1 µM) (n = 3). **(C)** Caspase 3/7 activity and **(D)** viability of C28/I2 cells treated for 8 h with pH 7.4, pH 5.5 and pH 7.4 + DIDS (100 µM) in the absence or presence of staurosporine (st) (1 and 5 µM) (n = 4). **(E)** Caspase 3/7 activity and **(F)** viability of cells treated for 24 h with pH 7.4 and pH 6.0 with or without DCPIB (10 µM) in the absence or presence of staurosporine (st) (1 µM) (n = 2–3). Data are represented as mean ± SEM.

To test whether the protective effect of low pH in C28/I2 cells is associated with VSOR channel activity, we investigated the effects of Cl^−^ channel inhibitors on staurosporine-induced apoptosis. NFA (500 µM), NPPB (100 µM) and DIDS (100 µM) attenuated apoptosis induced by 1 µM staurosporine, as evidenced by a decreasing caspase 3/7 activity and an increasing viability compared to controls in the absence of the VSOR Cl^−^ channel inhibitors (Figures 6A–D). However, while the reduction in caspase activity under staurosporine was comparable between the three inhibitors and exposure to pH 5.0, the increase in viability was less evident for NFA and NPPB as compared to DIDS and pH 5.0. DCPIB (5 or 10 µM) did not affect caspase 3/7 activity and viability at pH 7.4 in the absence or presence of staurosporine (Figures 6A,B) but caused a decrease in staurosporine-induced caspase activity and a strong reduction in cell viability at pH 6.0 (Figures 6E,F). Tamoxifen at 10 µM showed similar effects as DCPIB on caspase activity in both absence and presence of staurosporine (Figure 6A) but caused a drastic decline in cell viability in the presence of the apoptosis inducer (Figure 6B). In addition, annexin-V/7-AAD double staining revealed that DIDS (100 µM) increased the Q4 annexin-V–/7-AAD– (living) cell population by ∼20% and decreased the Q3 annexin-V+/7-AAD– (early apoptotic) cell population of ∼20% after staurosporine treatment (1 µM) compared to cells treated with staurosporine alone in the absence of DIDS (pH 7.4 + st (1 µM)) (Figures 4C,D and Figure 5F). DIDS had no effect on the Q4 annexin-V–/7-AAD– (living) and Q3 annexin V+/7-AAD– (early apoptotic) cell populations, when cells were exposed to 5 µM staurosporine (Figures 4C,D). In comparison to control (pH 7.4), DIDS alone had no effect on the Q1–Q4 distribution of annexin-V/7-AAD double stained cells (Figures 4A–D and Figure 5E).

### Acidic Conditions and Volume-Sensitive Outwardly Rectifying Cl^−^ Channel Inhibitors Prevent Staurosporine-Induced Normotonic Cell Shrinkage

**FIGURE 7:**
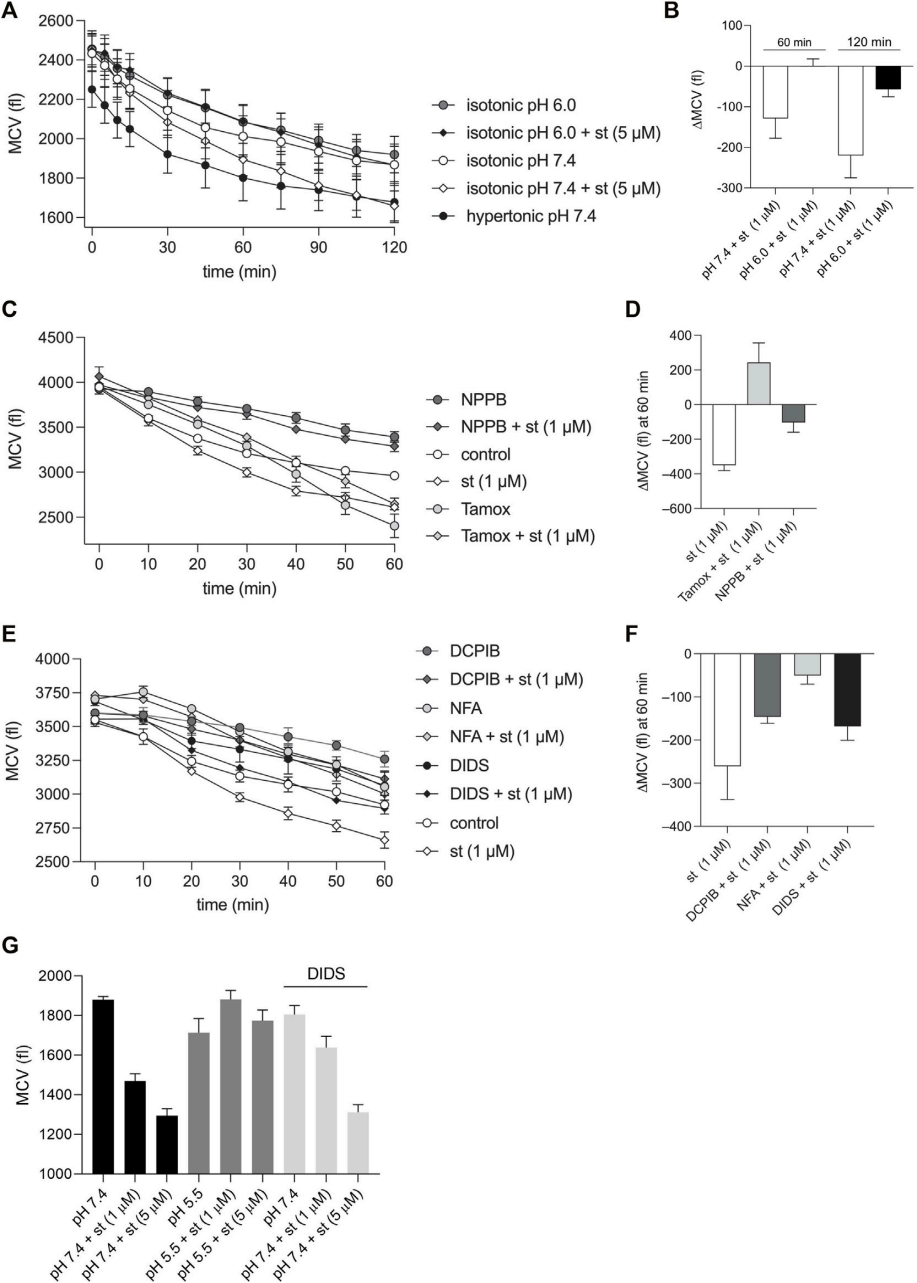
AVD in chondrocytes is impaired under acidic conditions and in the presence of VSOR Cl-channel inhibitors. **(A, C, E)** Mean cell volume (MCV) ± SEM in femtoliters (fl) measured **(A)** over 2 h under hypertonic (360 mOsm/kg) pH 7.4 conditions and under isotonic (300 mOsm/kg) conditions at pH 7.4 or pH 6.0 in the presence and absence of 5 µM staurosporine (st) (n = 4) and **(C, E)** MCV measured over 60 min under isotonic pH 7.4 conditions in the absence (control) or presence of the Cl^−^ channel inhibitors NPPB (100 µM), Tamoxifen (Tamox) (10 µM), DCPIB (10 µM), niflumic acid (NFA; 500 µM) or DIDS (100 µM) ± staurosporine (1 µM) (n = 3). **(B)** Differences in MCV (ΔMCV in fl) calculated from data in **(A)** at the timepoints 60 and 120 min for matching pH conditions (pH 7.4 and 6.0) in the presence and absence of staurosporine. **(D, F)** ΔMCV at 60 min calculated from data in **(C, E)** for control (absence of blockers) and matching blocker conditions in the presence and absence of 1 µM staurosporine. **(G)** MCV ± SEM assessed by using flow cytometry after 5 h of incubation with pH 7.4, pH 7.4 + DIDS (100 µM) and pH 5.5 in the presence and absence of staurosporine (st) (1 µM or 5 µM) (n = 4).

As shown in Figure 7A, the exposure of C28/I2 cells to staurosporine (5 µM) resulted in a reduction of the MCV. Application of a hypertonic solution (360 mOsm/kg) also induced cell shrinkage, but in contrast to the staurosporine-induced cell shrinkage, the decline in the cell volume was more pronounced at earlier timepoints and flattened after ∼30 min, suggesting the induction of a regulatory volume increase (RVI) response. The staurosporine-induced AVD was completely abolished under pH 6.0. After 60 and 120 min under isotonic pH 7.4 + staurosporine the MCV was ∼-120 and -250 fl lower compared to cells kept under isotonic pH 7.4 (Figure 7A and depicted as ΔMCV in Figure 7B), whereas at pH 6.0 there was no difference measured after 60 min and after 120 min the difference in MCV was only ∼-50 fl. In further experiments we tested for the effects of Cl^−^ channel blockers on the time course of staurosporine-induced AVD. These experiments were performed under isotonic conditions and pH 7.4. On average, staurosporine induced an AVD (ΔMCV) of ∼-300 fl over 60 min *versus* control (Figures 7C–F). All tested inhibitors but Tamoxifen reduced the AVD after 60 min between cells kept in the absence and presence of staurosporine. Under NPPB (100 µM), DCPIB (10 µM), NFA (500 µM) and DIDS (100 µM) the ΔMCV was ∼-100, -150, -50 and -150 fl, respectively. Cells kept under 10 µM Tamoxifen showed a strong decline in the MCV after 30 min and signs of cell death under the microscope (not shown). After 60 min the MCV was higher in cells exposed to Tamoxifen + staurosporine as compared to cells in presence of Tamoxifen alone (Figures 7C,D). By using flow cytometry, we assessed the MCV after 5 h. The data show that the AVD induced by 1 µM (cell shrinkage from ∼1,850 to ∼1,450 fl) and 5 µM staurosporine (cell shrinkage from ∼1,850 to ∼1,300 fl) was completely abolished under pH 5.5 and was attenuated by the Cl^−^ channel inhibitor DIDS (100 µM) at 1 µM (cell shrinkage from ∼1,800 to ∼1,620 fl) but not at 5 µM staurosporine (Figure 7G).

## Discussion

**FIGURE 8:**
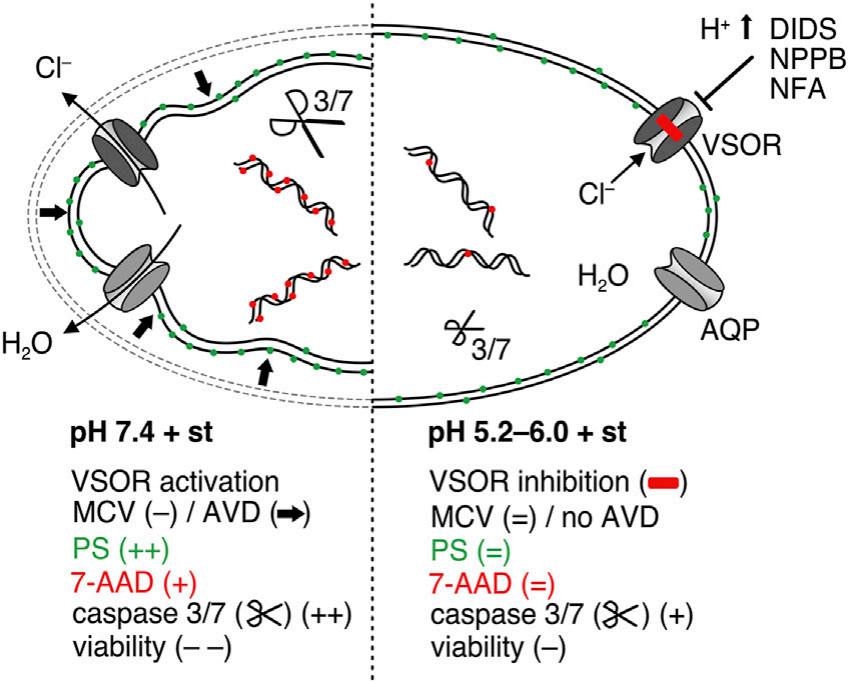
Putative model of the antiapoptotic effect of acidosis in C28/I2 cells treated with staurosporine. Under pH 7.4 (left side) staurosporine (st) treatment leads to activation of volume-sensitive outwardly rectifying (VSOR) Cl^−^ channels to induce cell shrinkage (AVD), which is an initial step during apoptosis, followed by phosphatidylserine (PS) exposure, caspase activation, DNA fragmentation, membrane blebbing and loss of membrane integrity evidenced by the 7-AAD uptake. Under acidic conditions (right side) VSOR channels are deactivated so that AVD is absent under isotonic conditions and consequently apoptosis is suppressed in the presence of staurosporine.

The results of the present study show that extracellular acidosis deactivates VSOR channels and protects chondrocytes from apoptosis. Specifically, we demonstrated that low pH reduces the caspase 3/7 activity and increases the viability after exposing C28/I2 cells to the apoptosis inducer staurosporine, as illustrated in Figure 8. Although the protective effect of acidosis against apoptosis has been shown in different cell types, very little is known about the underlying mechanisms. However, Ca^2+^ signaling, tyrosine kinase receptor Axl expression and mitochondrial ATP production have been suggested to play a role in the protective effect of acidosis (D'Arcangelo et al., 2002; Terminella et al., 2002; Sharma et al., 2005). E.g., in osteoclasts, the protective effect of acidosis against apoptosis was shown to be mediated by a rise in the intracellular Ca^2+^ concentration through proton-sensing receptor ovarian cancer G protein-coupled receptor 1 (OGR1) signalling (Pereverzev et al., 2008). To the best of our knowledge, this is the first report that VSOR currents are likely to be important for the acidosis-induced antiapoptotic effect in chondrocytes.

We show that lowering the pH to 6.0 leads to gradual deactivation of the volume-sensitive outwardly rectifying (VSOR) Cl^−^ current. Immediately after lowering the pH, an accelerated time-dependent inactivation at high positive potentials was observed, which is in line with earlier results obtained in Xenopus oocytes (Ackerman et al., 1994), C6 glioma cells (Jackson and Strange, 1995), BC3H1 mouse myoblasts (Voets et al., 1997), or bovine pulmonary artery cells (Nilius et al., 1998) and might be interpreted as the onset phase of acid-induced VSOR current deactivation. In addition, we showed recently in chondrocytes and microglial cells that a strong acidification to pH ≤ 5.0 causes a fast and complete deactivation of the VSOR current and simultaneous activation of an acid-sensitive outwardly rectifying (ASOR) Cl^−^ current (also termed proton-activated Cl^−^ current/channel; PAC or PACC) (Kittl et al., 2019a; Kittl et al., 2020). To prevent ASOR current activation, in the present study we therefore avoided acidification below pH 5.0. The mechanism underlying acidity-induced deactivation of the VSOR Cl^−^ current over time is unknown. It might be caused by conformational changes of the channel protein itself after proton binding, altered interaction with regulatory factors, or altered expression of channel entities. Until recently, the molecular identity of the VSOR channel remained obscure and has been controversially debated (Pedersen et al., 2015). Meanwhile there is compelling evidence from gene-silencing studies that leucine-rich repeat containing 8 (LRRC8) family members A–E are essential components of VSOR channels (Pedersen et al., 2015; Jentsch, 2016; Jentsch et al., 2016), but it is still possible, that additional proteins are involved in channel formation and/or regulation of VSOR channel activity (Okada et al., 2020). With respect to long-term exposure to staurosporine or hypoosmotic conditions, previous studies suggest that changes in VSOR channel activity are more likely caused by modulation of the function of LRRC8 channel proteins rather than by their abundance (Bach et al., 2018; Yurinskaya et al., 2020). To date there is no information available on the pH-sensitivity of LRRC8 proteins on the expressional and/or functional level. This needs to be addressed in future studies.

Apoptotic volume decrease (AVD) is an early event in apoptosis, which starts before caspase activation and DNA fragmentation and is characterized by a normotonic shrinkage of cells (Okada and Maeno, 2001; Elmore, 2007; Poulsen et al., 2010; Maeno et al., 2012; Okada et al., 2020). The inhibition of the VSOR Cl^−^ current has been shown to prevent drug-induced AVD and the following steps of apoptosis induced by activation of either the intrinsic or extrinsic pathway (Maeno et al., 2000; Okada et al., 2001; Ernest et al., 2008). Specifically, the reduction in the intracellular Cl^−^ concentration during AVD is essential for the activation of DNA fragmentation factors (Rasola et al., 1999; Dupere-Minier et al., 2004). In the present study, staurosporine induced an AVD within 30 min, which was completely absent under acidic conditions (pH 6.0 and 5.5). We also observed that not only the AVD but also the RVD was impaired by acidity, which supports the assumption, that the induction of AVD under normotonic conditions and RVD under hypotonic conditions share common volume regulatory mechanisms including VSOR Cl^−^ current activation (Maeno et al., 2000). Furthermore, an involvement of the VSOR Cl^−^ channel in AVD is supported by the findings that 1) AVD was abolished by pharmacological inhibition of Cl^−^ channels and (Maeno et al., 2000; Okada et al., 2006), 2) inducers of apoptosis like staurosporine, doxorubicin, FAS ligand, TNFα, or H_2_O_2_ activate VSOR Cl^−^ channels (D’anglemont De Tassigny et al., 2004; Porcelli et al., 2004; Shimizu et al., 2004; Jiao et al., 2006) and 3) apoptotic cells undergoing AVD display RVD facilitation (Maeno et al., 2000). In a study on salmonid hepatoma and gill cells, DIDS, an inhibitor of Cl^−^ channels, partially suppressed AVD (Krumschnabel et al., 2007), while Maeno et al. and Jiao et al. observed a complete suppression (Jiao et al., 2006; Maeno et al., 2012). In addition, Cl^−^ channel blockers like NPPB and phloretin have also been reported to inhibit AVD completely whereas SITS, niflumic acid (NFA) and glibenclamide have been shown to attenuate AVD. In line with these previous findings, in our present work we observed a reduction in the AVD response in C28/I2 cells by DIDS, NPPB, NFA and DCPIB. The same blockers have also been described to prevent ischemia-induced, serum deprivation-induced and drug-induced apoptotic cell death in various cells including endothelial cells (D’Arcangelo et al., 2000; D'Arcangelo et al., 2002; Kumar et al., 2008), liver cells (Currin et al., 1991), lymphoblastic cells (Bohloli et al., 2016), neurons (Xu et al., 1998) and tumor cells (Akbar and Kim, 2002; Sharma et al., 2005). In the present volume experiments, we observed a cell shrinkage over time under isotonic conditions and normal pH, which has similarly been described in human epithelial cells, cortical neurons, insulinoma cells and microglial cells (Jakab et al., 2006; Wang et al., 2007; Sato-Numata et al., 2014; Kittl et al., 2019a). As already discussed in our previous paper (Kittl et al., 2019a), this might be caused by an imbalance in osmolality under the given conditions with relative hypertonicity of the extracellular solution.

Cl^−^ channel blockers have also been found to reduce apoptosis in cells treated by the anti-cancer drug cisplatin (Cai et al., 2015). Only a few studies challenge the concept that AVD is an early prerequisite of apoptosis leading to cell death, by showing that VSOR Cl^−^ channel blockers inhibited AVD without preventing apoptotic cell death. For example, in RAW 264.7 macrophages and cortical neurons, VSOR channel blockers prevented AVD, whereas apoptotic cell death was not or only mildly attenuated (Hortelano et al., 2002; Wei et al., 2004). Our study in human chondrocytes provides evidence, that acidosis has a similar anti-apoptotic effect like pharmacological inhibition of VSOR Cl^−^ channels. We assume that acidosis gradually deactivates VSOR Cl^−^ currents, which—by preventing AVD—impedes the progression of apoptosis evidenced by a higher viability in chondrocytes after staurosporine treatment (Figure 8). Interestingly, in the present study pharmacological inhibition of VSOR Cl^−^ current with DIDS, NPPB and NFA was less protective than acidosis, indicating that the protective effect of acidosis in chondrocytes is not solely based on the inhibition of Cl^−^ currents. A major shortcoming of Cl^−^ channel blockers in their commonly used concentrations is their lack in specificity, which compromises their explanatory potential. VSOR channel inhibitors like DIDS and NPPB also block other cell volume-correlated anion channels such as Maxi-Cl^-^ channels and acid-sensitive outwardly rectifying (ASOR) channels (Friard et al., 2017; Kittl et al., 2019a; Kittl et al., 2020; Okada et al., 2021). DCPIB has also been shown to inhibit K^+^ channels involved in cell volume regulation such as TWIK and TASK K2P channels (Lv et al., 2019).

Indeed, a number of studies have linked apoptosis not only to Cl^−^ channels but also to other ion channels (Hoffmann et al., 2009). K^+^ and Na^+^, critical determinants of cell volume, have been proposed to play a role in apoptosis. For example, the K^+^ channel blockers tetraethylammonium (TEA) and clofilium effectively attenuated AVD as well as apoptotic cell death in cortical neurons (Wei et al., 2004). Also, intracellular Ca^2+^ is tightly linked to apoptosis. As an example, it has been shown that a cytoplasmic Ca^2+^ overload by increasing the L-type Ca^2+^ channel activity triggers apoptosis in endocrine cells (Juntti-Berggren et al., 1993). In contrast, the inhibition of T-type Ca^2+^ channels has been reported to induce p53-dependent apoptosis in colon cancer cells (Dziegielewska et al., 2014). Some of the channels, which are involved in apoptosis, are known to be pH sensitive (Delisle and Satin, 2000; Petho et al., 2020), which might add to the anti-apoptotic effect of low pH explaining the strong anti-apoptotic effect of acidosis and a weaker effect of Cl^−^ channel inhibition alone, as observed in our present study. In addition, the VSOR Cl^−^ channel inhibitors DCPIB and Tamoxifen were used in the present study to test their effect on apoptosis. Surprisingly, our data suggest that these two inhibitors promote cell death after staurosporine treatment under both acidic and pH 7.4 conditions, while in a study on cardiomyocytes by Wang et al., DCPIB like DIDS inhibited apoptosis and restored viability after treatment with high glucose (Wang et al., 2017). It is important to consider that DCPIB might have significant off-target effects. A recent study demonstrated that DCPIB suppresses mitochondrial respiration independently of VSOR Cl^−^ channel activity (Afzal et al., 2019). We suggest that the exposure to 10 µM DCPIB or Tamoxifen for several hours might similarly impair the viability of chondrocytes and promote cell death by exerting cytotoxic effects.

Tissue areas of locally decreased pH are hallmarks of many diseases or disease-associated conditions. By using surface enhanced Raman spectroscopy, high concentrations of protons could be visualized locally at the cell membrane of gastric tumor cells, with a low surface interstitial pH of 6.0 (Puppulin et al., 2018). Low local pH could also be detected under pathological conditions like ischemia (Smith et al., 1986), or atherosclerotic plaques (Naghavi et al., 2002) with pH values of ∼6.5 and ∼7.0, respectively. Patients with rheumatoid arthritis showed significantly lower pH values of the synovial fluid than healthy subjects (Cummings and Nordby, 1966) and in patients with primary hip OA, intraoperative *in situ* pH measurements revealed massive acidification at the cartilage surface to values as low as ∼5.5 at stage 3 (Konttinen et al., 2002). In this pH-range we found reduced apoptotic cell death and a promotion of the survivability of chondrocytes. *In vivo*, this might contribute to the appearance of hypercellularity of chondrocytes observed in early stages of OA (Galois et al., 2004; Pauli et al., 2011) and in the subsequent degradation in advanced states of OA (Hwang and Kim, 2015). During aging, the number of chondrocytes also increases in the deep layer of cartilage, leading to OA by subchondral tissue calcification or other structural changes of the cartilage (Akkiraju and Nohe, 2015). Interestingly, in one experimental series of the present study shown in Figure 3B, we observed a slightly higher viability under acidic conditions ≥ pH 5.5. With respect to cell volume, in an anterior cruciate ligament transection (ACLT) rabbit osteoarthritis model, chondrocytes have shown to be swollen in comparison to controls. Kumagai et al. concluded that this phenomenon was due to a reduced capacity of RVD (Kumagai et al., 2016). Importantly, we show here and in a previous study that cell volume regulation in chondrocytes is impaired under acidic conditions (Kittl et al., 2020). Taken together, impaired RVD, prevention of AVD and apoptosis and a higher survivability (hypercellularity) in chondrocytes under acidic conditions may possibly play a role in the onset and/or development of OA.

In summary, this study shows that VSOR Cl^−^ channels are inhibited under acidic conditions. Since Cl^−^ channels are involved in volume regulatory processes like RVD and AVD, cell volume homeostasis and apoptosis are massively deranged, and the viability of chondrocytes is enhanced by acidity. Given that the maintenance of extracellular matrix production and cartilage integrity exclusively depends on the functional stability of chondrocytes, compromised cell volume homeostasis and altered viability are likely to promote articular cartilage degeneration and progression of OA.

## Data Availability

The original contributions presented in the study are included in the article/Supplementary Material, further inquiries can be directed to the corresponding author.
